# Quantitative bone scan lesion area as an early surrogate outcome measure indicative of overall survival in metastatic prostate cancer

**DOI:** 10.1117/1.JMI.5.1.011017

**Published:** 2018-01-11

**Authors:** Matthew S. Brown, Grace Hyun J. Kim, Gregory H. Chu, Bharath Ramakrishna, Martin Allen-Auerbach, Cheryce P. Fischer, Benjamin Levine, Pawan K. Gupta, Christiaan W. Schiepers, Jonathan G. Goldin

**Affiliations:** a University of California Los Angeles, Center for Computer Vision and Imaging Biomarkers, Los Angeles, California, United States; b University of California Los Angeles, Department of Radiological Sciences, Los Angeles, California, United States; c University of California Los Angeles, Department of Nuclear Medicine, Los Angeles, California, United States

**Keywords:** prostate cancer, bone scan, computer-aided diagnosis

## Abstract

A clinical validation of the bone scan lesion area (BSLA) as a quantitative imaging biomarker was performed in metastatic castration-resistant prostate cancer (mCRPC). BSLA was computed from whole-body bone scintigraphy at baseline and week 12 posttreatment in a cohort of 198 mCRPC subjects (127 treated and 71 placebo) from a clinical trial involving a different drug from the initial biomarker development. BSLA computation involved automated image normalization, lesion segmentation, and summation of the total area of segmented lesions on bone scan AP and PA views as a measure of tumor burden. As a predictive biomarker, treated subjects with baseline BSLA $<200\;{\mathrm{cm}}^{2}$ had longer survival than those with higher BSLA (HR=0.4 and p<0.001). As a surrogate outcome biomarker, subjects were categorized as progressive disease (PD) if the BSLA increased by a prespecified 30% or more from baseline to week 12 and non-PD otherwise. Overall survival rates between PD and non-PD groups were statistically different (HR=0.64 and p=0.007). Subjects without PD at week 12 had longer survival than subjects with PD: median 398 days versus 280 days. BSLA has now been demonstrated to be an early surrogate outcome for overall survival in different prostate cancer drug treatments.

## 1 Introduction

More than 90% of patients with advanced prostate cancer develop bone metastases,^1^ which can produce some of the most severe complications of the disease and is associated with shorter survival times.^2^^–^^6^ New drugs are under development for metastatic castration-resistant prostate cancer (mCRPC), and there is a need for biomarkers to identify target populations and for early evaluation of treatment effects as an alternative to overall survival, which leads to long studies and is becoming problematic due to subject crossover and contamination from multiple therapies.

Whole-body bone scintigraphy is the accepted standard imaging modality for detection of bone metastases and assessment of treatment outcomes. Response evaluation criteria in solid tumors, the standard guideline used to assess outcomes in solid tumor malignancies, treats bone lesions as “nonmeasurable” and is therefore of limited usefulness in the setting of prostate cancer treatments.^7^ Therefore, the Prostate Cancer Working Group 2 (PCWG2) developed visually based criteria for assessing disease progression on bone scans based on counting new lesions.^8^ PCWG2 does address the significance of changes in intensity, size, or area of individual lesions, all of which are limited by the challenges of subjective, visual lesion detection. The simple conventional metric of lesion counting is of limited value when assessing treatment effects, as lesions may decrease in size without changing in number or may break into smaller components and, thus, superficially appear as an increased metastatic burden. This motivated the development of computer-aided quantitative measures of disease burden on bone scans.

The two quantitative bone imaging biomarkers that have undergone the most study and use in oncologic clinical trials are the bone scan index (BSI)^9^ and bone scan lesion area (BSLA).^10^ The BSI sums the product of the estimated weight and the fractional involvement of each bone, determined visually or from lesion segmentation on the bone scan. BSI was first evaluated as a prognostic biomarker^11^ and has had a number of more recent follow-up studies.^12^^,^^13^ BSLA was the first quantitative imaging biomarker to be developed and evaluated primarily for treatment response assessment in prostate cancer. The calculation and its ongoing clinical validation will be described in this paper.

In development of an imaging biomarker, there are two important phases: (1) development and analytic validation (including training of classifiers, determination of cut points, assessment of reproducibility, and evaluation against radiologist measurements) and (2) clinical validation in which the system and its cut points are fixed and it is evaluated against outcomes in new clinical trial data. BSLA is an imaging biomarker computed from whole-body scintigraphic imaging as a measure of overall bone tumor burden. Initial development and analytic validation, including evaluation against manual tumor segmentation^14^ and determination of response thresholds using trial cohorts, are from a single drug treatment (cabozantinib) with controls in subjects with metastatic CRPC.^10^^,^^15^ A 30% increase/decrease in BSLA relative to baseline was defined as progression/response on bone scan based on the data from these previous cohorts. Because of the promising results and urgent need, the BSLA imaging biomarker was rapidly adopted in clinical trials, such as^16^ using the drug for which the biomarker was initially developed and, in Ref. 17, an ongoing trial using a different treatment. Therefore, rather than an investigation into threshold or other algorithm parameters, this paper is focused on clinical validation of an existing test that has been adopted by the research community in prostate cancer clinical trials. In the computer-aided diagnosis research community, the majority of papers involve analytic validation, and this clinical validation is the next step in putting biomarker translation into practice. As a clinical validation, the analysis approach in this paper is consistent with those used in clinical trials.

We seek to establish the clinical value of an existing imaging test based on the quantitative BSLA and will investigate and evaluate the biomarker as a prognostic factor, predictive factor, and surrogate outcome marker. A prognostic factor is a clinical or biologic characteristic that is objectively measurable and that provides information on the likely outcome of the cancer disease in an untreated individual. A predictive factor is a clinical or biologic characteristic that provides information on the likely benefit from treatment (either in terms of tumor shrinkage or survival). Such predictive factors can be used to identify subpopulations of patients who are most likely to benefit from a given therapy. Importantly, prognostic factors define the effects of patient or tumor characteristics on the patient outcome, whereas predictive factors define the effect of treatment on the tumor.^18^ A surrogate outcome marker can be defined as a laboratory measurement that is used in therapeutic trials as a substitute for a clinically meaningful endpoint, such as survival, and is expected to predict the effect of the therapy.^19^^,^^20^

We hypothesize that, when applied to an independent treatment trial cohort with a different mechanism of drug action, a week 12 change posttreatment using this prespecified threshold for progression is predictive of a subject’s overall survival, i.e., can be used as a surrogate outcome marker. Second, we evaluated the potential of baseline BSLA (disease burden on the baseline scan) as a predictive biomarker used to identify patients most likely to benefit from treatment.

## 2 Methods

### 2.1 Data Collection

From an anonymized imaging research database, a cohort of 198 mCRPC subjects who enrolled in a multicenter treatment trial of abiraterone acetate (127 treated and 71 placebo) using a standardized imaging protocol was identified. Subjects were included that had whole-body original DICOM images and survival data available. This cohort was independent of those used for development of the biomarker criteria for progression/response and involved a different mechanism of drug action. Subjects underwent the standard of care whole-body bone scintigraphy with 99mTc-Methyl diphosphonate (99mTc-MDP) at baseline and week 12 posttreatment.

### 2.2 Bone Scan Image Processing

A CADrx system for bone scan assessment was developed within the imaging biomarker information system (IBIS) for image markup and analysis (MedQIA, LLC, Los Angeles, California). The IBIS markup system combines image review capabilities with computer-aided tools for region segmentation, quantitative analysis, and data export for clinical trials. In the CADrx system, anterior and posterior bone scan images are processed with pixel intensity normalization and lesion segmentation, followed by quantitative assessment of lesion burden. The image analysis method was previously described in detail,^10^^,^^21^ and the steps are summarized here.

#### 2.2.1 Anatomical region segmentation

**Fig. 1:**
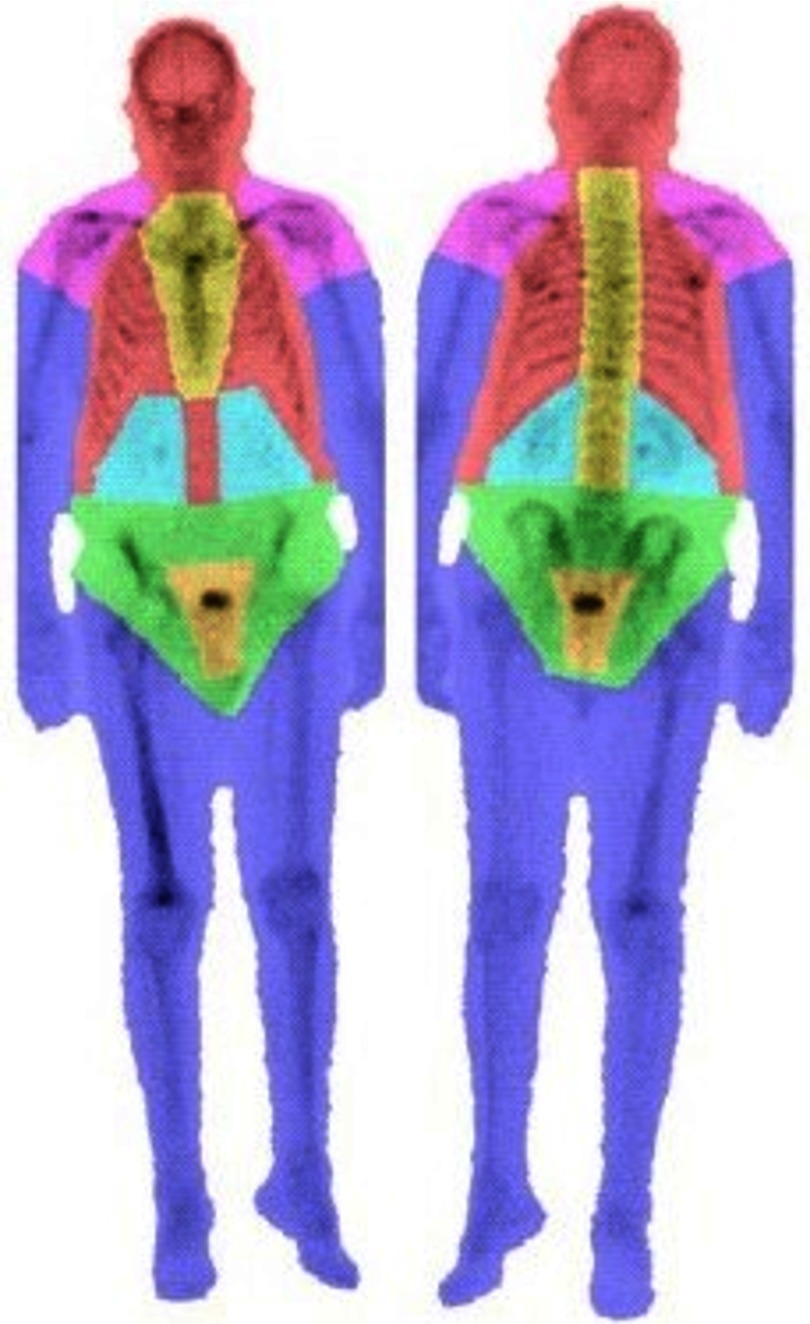
Automated anatomical region segmentation of ribs/head (red), spine/sternum (yellow), pelvis (green), extremities (blue), shoulders (magenta), kidney search region (cyan), and bladder search region (orange).

Atlas-based segmentation was performed to label seven anatomical regions on the bone scan: sternum/spine, ribs/head, extremities (arms and legs), pelvis, shoulders, kidney search region, and bladder search region. Registration to the atlas involved affine registration using the Mattes mutual information metric^22^ followed by a multiresolution demons deformable registration.^23^ An example of the output of the anatomical region segmentation is shown in Fig. 1.

#### 2.2.2 Image intensity normalization

Image intensity normalization was applied to reduce inter- and intrapatient variations due to differences in body habitus, radiotracer dosing levels, and scan acquisition parameters. A region of normal bone in the extremities was identified automatically based on the anatomical region segmentation. Then all pixel values were linearly rescaled to set the intensity of this normal bone to a reference intensity. After normalization, the pixel intensities of normal bone are consistent between subjects and across time points for a given subject, allowing for reproducible lesion segmentation and quantitative assessment in serial patient images.

#### 2.2.3 Automated lesion detection

Based on the atlas-based segmentation, anatomical region-specific lesion intensity thresholds for lesion detection were learned previously using receiver operating characteristic (ROC) analysis on a training set of images.^10^ The ROC analysis was used to set the region-specific thresholds to maximize segmentation accuracy against expert delineated reference segmentations on the training set.^14^ An additional classification stage was applied to candidate lesions generated by the thresholding to remove false positives related to bladder uptake, kidney uptake, and symmetric degenerative joint disease.^21^ False positives related to uptake in these anatomical regions were identified and removed based on overlap with corresponding regions from the atlas-based segmentation. Symmetric degenerative joint disease removal involved computing features of lesion candidates: lesion area, mean intensity, perpendicular distance from the midline, and vertical distance along the midline. Lesion candidates were compared in a pairwise manner and symmetric pairs identified based on feature difference thresholds. Parameters in the false positive reduction were trained using a multistart local optimization method using the Nelder–Mead simplex.^24^

#### 2.2.4 Segmentation review and approval

For each bone scan, the results of the automated lesion segmentation were reviewed by a nuclear medicine physician and manually edited (lesion pixels added or removed) as needed. This editing typically involved removal of any remaining false-positive regions (e.g., areas of degenerative joint disease) and took on the order of minutes per scan. Previous studies showed 89% pixel accuracy of the lesion segmentation method against manual expert annotations, so the amount of editing required for a given case is typically minimal.^14^

### 2.3 Treatment Response Assessment Using Bone Scan Lesion Area

**Fig. 2:**
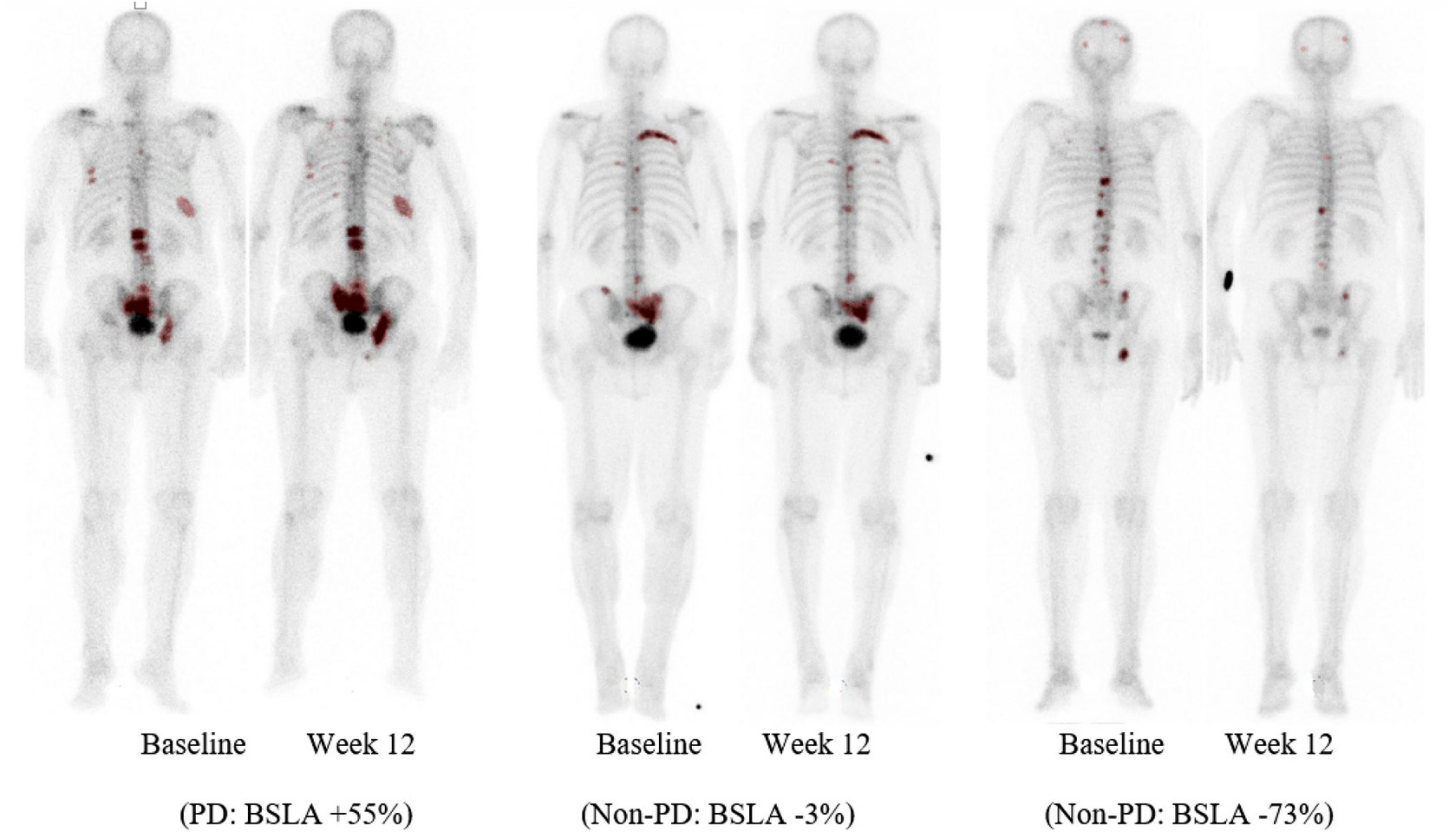
Example cases with lesions segmented in red and BSLA change assessment.

BSLA is summed as $\mathrm{BSLA}=\sum_{p\in R}A_{p}$, where R is the set of pixels identified as bone lesion and $A_{p}$ is the physical area of pixel p (in ${\mathrm{cm}}^{2}$). The BSLA measure thus represents a quantification of the size and number of active regions on the bone scan. BSLA was calculated at baseline and week 12 posttreatment for all subjects in the study. A prespecified 30% increase in BSLA from baseline to week 12 was used to identify subjects with progressive disease (PD). Subjects with <30% increase or decrease in BSLA were categorized as nonprogressive disease (non-PD). For evaluation as a prognostic factor, the dataset was dichotomized about the median baseline BSLA. Figure 2 shows examples of bone scans with lesions semiautomatically segmented in red and changes in BSLA computed from baseline to week 12. The examples reflect PD (an increase in lesion burden) and non-PD (stable and reduction in lesion burden).

### 2.4 Statistical Analysis

BSLA was evaluated as a prognostic factor, predictive biomarker, and a surrogate outcome biomarker. Subjects were grouped as PD versus non-PD and multivariate Cox regression was used to test whether (1) baseline BSLA and (2) early changes in BSLA (12 weeks posttreatment) were predictive of overall survival. Landmark survival analyses were used to assess early changes. Kaplan–Meier plots and hazard ratios were used to evaluate differences among groups defined by the BSLA biomarker.

## 3 Results

### 3.1 Prognostic and Predictive Biomarker Evaluation

**Fig. 3:**
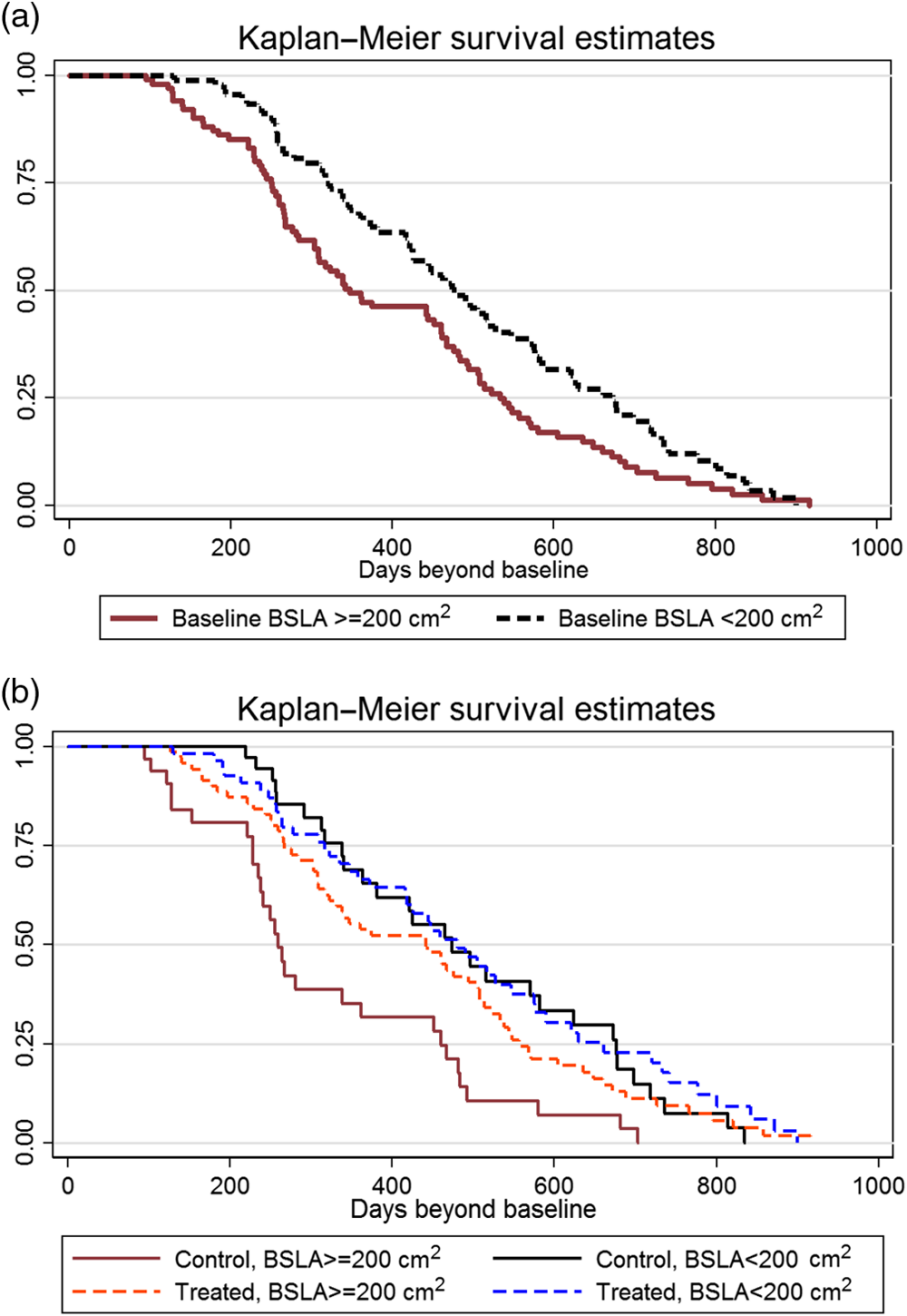
Kaplan–Meier plots for low BSLA ($<200\;{\mathrm{cm}}^{2}$) versus high BSLA ($>=200\;{\mathrm{cm}}^{2}$) as (a) prognostic factor and (b) predictive biomarker.

Median BSLA at baseline was $219\;{\mathrm{cm}}^{2}$. BSLA $<200\;{\mathrm{cm}}^{2}$ at baseline was a prognostic factor for delaying progression (HR=0.6 and p=0.003) and predictive of longer survival (HR=0.4 and p<0.001). Figure 3 shows Kaplan–Meier plots of the proportion of subjects surviving a given number of days beyond baseline when separated into groups based on the baseline BSLA score.

Figure 3(a) shows BSLA as a prognostic factor including all subjects, both treatment and control groups. It shows that subjects with low baseline BSLA scores ($<200\;{\mathrm{cm}}^{2}$) have a better overall prognosis in terms of survival time.

Figure 3(b) shows BSLA as a predictive biomarker with subjects separated into treatment and control groups. It shows that subjects with high baseline BSLA scores ($>=200\;{\mathrm{cm}}^{2}$) can be predicted to experience treatment benefit relative to controls (red versus brown survival curves). Subjects with low baseline BSLA scores undergoing treatment ($<200\;{\mathrm{cm}}^{2}$) can be predicted to have a better survival outcome than those with high BSLA scores. Subjects with low baseline BSLA scores have a relatively good overall prognosis, irrespective of whether they are treated (blue and black survival curves).

### 3.2 Early Surrogate Outcome Evaluation

**Fig. 4:**
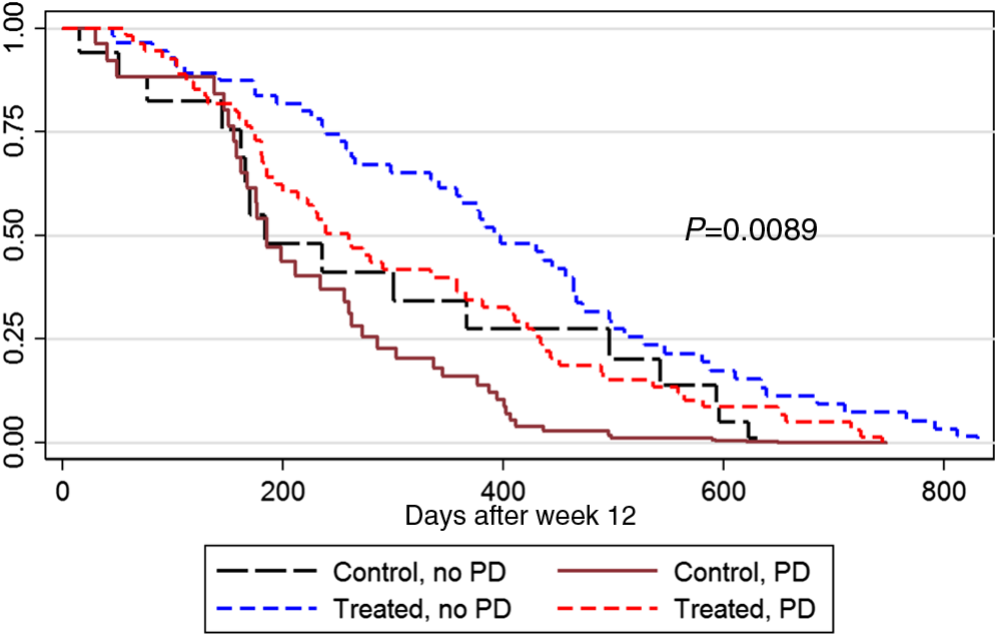
Survival plots for non-PD versus PD by BSLA at week 12 in control (placebo) and treatment groups after adjustment of baseline BSLA score $<200\;{\mathrm{cm}}^{2}$.

Overall survival rates between PD and non-PD groups were statistically different (HR=0.64 and p=0.007). Subjects without PD by BSLA at week 12 had longer survival than subjects with PD: median 398 days versus 280 days (378 days versus 228 days after adjustment for baseline BSLA $<200\;{\mathrm{cm}}^{2}$). Similar differences were seen within the treatment and placebo groups (see Table 1). The corresponding Kaplan–Meier survival curves are shown in Fig. 4, and multivariate Cox regression analysis for survival is shown in Table 2.

**Table 1:** Median survival in days after week 12, with number of subjects in each group (adjusting for baseline BSLA score $<200\;{\mathrm{cm}}^{2}$).

Median (±IQR) survival after week 12 (N=number of subjects)	PD by BSLA	Non-PD by BSLA
Placebo group	186 (±221) days (N=45)	170 (±222) days (N=26)
Treatment group	260 (±254) days (N=65)	392 (±311) days (N=61)
All	228 (±242) days (N=111)	378 (±327) days (N=87)

**Table 2:** Multivariate Cox regression for survival.

Coefficient	HZ (±SE)	p-value	95% CI
Treatment	0.49 (±0.11)	0.002	[0.32, 0.76]
Baseline BSLA $<200\;{\mathrm{cm}}^{2}$	0.34 (±0.09)	<0.001	[0.20, 0.58]
Interaction between treatment and BSLA	2.15 (±0.70)	0.019	[1.14, 4.08]
Non-PD	0.64 (±0.11)	0.007	[0.46, 0.88]

## 4 Discussion

As a prognostic and predictive biomarker, the BSLA can facilitate patient management and prospective determination of those most likely to benefit from a given therapy, rather than beginning a therapy and waiting months to see if the disease progresses or not, which is particularly problematic for advanced prostate cancer. Specifically, subjects with high BSLA should be treated (low BSLA has a relatively good prognosis regardless of whether treated or not). In addition to using the median baseline BSLA (50th centile) as a prognostic cut point, we performed a sensitivity analysis by testing the 33rd and 67th centiles. Both of these also gave statistical significance as a prognostic factor, indicating that definitions of “low” and “high” baseline BSLA are likely to be robust.

Table 2 shows that treatment and non-PD are factors in lowering the hazard ratio, i.e., having longer survival. This is reflected in the Kaplan–Meier curves of Fig. 4; the treated, non-PD subjects have the longest survival times and the control, PD subjects the shortest. As a surrogate outcome measure, the BSLA can be used in clinical trials to speed up drug development by determining utility without waiting for survival. This can be particularly useful in adaptive designs and dose-ranging studies.^10^ It can thus be used to develop and evaluate new mCRPC therapies more quickly.

In this study, we dichotomized subjects based on PD only (i.e., PD versus non-PD, rather than responders versus nonresponders) to obtain similar numbers of subjects in each of the two groups. However, BSLA also allows classification of subjects as responders to therapy (reduction in BSLA of 30% or more) as described in Ref. 10. In a previous study,^15^ BSLA was used to group subjects into responders and nonresponders with significant differences hazard ratio (HR 0.47, 95% CI 0.28 to 0.79, and p=0.005). We did not form a separate responder group in this study since the number of such subjects was relatively low.

As described in the Introduction, BSI was originally evaluated as a prognostic biomarker. More recently, changes in BSI have also been investigated retrospectively, with various cut points for BSI groupings being explored rather than prespecified. In a mCRPC cohort, Reza et al.^25^ found that an increase in BSI at follow up of at most 0.30 had a significantly longer median survival time than those with an increase of BSI >0.30. They note that retrospective design (choice of BSI cut point) was a limitation. In another mCRPC cohort^26^ in which a different cut point of not >20% increase from BSI baseline was applied, they found that the group had a significantly longer time to progression in bone than those who had a BSI increase >20% during treatment. These studies differ from ours in that we prespecified the criteria for disease progression of 30% or more increase in BSLA and then applied it prospectively to this and other new cohorts to demonstrate robustness across different therapeutic protocols.

The focus of this paper has been on clinical validation of an existing algorithm already adopted in trials. However, as more data are becoming available, there will be an opportunity to update parameters in the algorithm, such as the intensity thresholds and response/progression cut points, and to include more advanced classifiers to further improve performance. For example, the currently used 30% cut point for progression/response was set conservatively in a small developmental set such that all control subjects had BSLA changes less than this threshold,^10^ and, as further reproducibility studies are performed, we may be able to reduce the threshold and increase the sensitivity of the biomarker. Because the initial developmental set was relatively small, the subsequent larger clinical validation studies, such as Ref. 16 and this one in a different drug treatment, are particularly important to show that the current algorithm in use in clinical trials provides an effective surrogate for overall survival.

## 5 Conclusion

BSLA is calculated semiautomatically from bone scans and provides a quantitative and objective treatment response assessment. Baseline BSLA and early changes posttreatment were found to be predictive of overall survival in patients with mCRPC. BSLA has now been demonstrated to be an early surrogate outcome for overall survival in different prostate cancer drug treatments.
